# Bootstrapping the early lexicon: how do children use old knowledge to create new meanings?

**DOI:** 10.3389/fpsyg.2013.00096

**Published:** 2013-03-04

**Authors:** Emily Mather

**Affiliations:** Department of Psychology, University of HullHull, UK

## Introduction

Compared to other aspects of language development, such as acquiring grammar, we perhaps take for granted the complexity of building a lexicon. More than 50 years ago the philosopher W.V.O. Quine provided a now famous example of what makes word learning so difficult. In short, Quine (1960) argues that when we hear a new word, how can we ever precisely determine its meaning? This problem has concerned many child language researchers, who all acknowledge that the child requires a means of narrowing down the infinite possible meanings of a word. However, researchers have advocated contrasting theoretical perspectives on how this problem is solved. For many, the child's ability to learn new words requires external input from the speaker—not just the word uttered, but also overt “clues” such as pointing and eye gaze (e.g., Bloom, 2000). Others argue that the child brings their own tools to the task, whether in the form of domain-specific heuristics (e.g., Golinkoff et al., 1994), or domain-general mechanisms (e.g., Samuelson and Smith, 2000).

One simple solution is to use existing vocabulary knowledge to decode the meanings of new words. This strategy is commonly known as *mutual exclusivity*—the assumption that an object can only have one name (Markman, 1989, 1990). If a child is faced with more than one possible referent of a new word, she will map the word to whichever referent they cannot name. Word learning in this manner is a form of “bootstrapping” in language development, where existing lexical knowledge can be exploited in order to acquire new lexical knowledge. Alternative explanations of this word learning behavior include the *novel-name-nameless-category* (N3C) *principle* (Mervis and Bertrand, 1994) and the *principle of contrast* (Clark, 1987). In spite of extensive research into mutual exclusivity (and related theoretical accounts), many aspects of this phenomenon are not well-understood, such as the underlying cognitive mechanisms and developmental origins.

### What mechanism underlies mutual exclusivity?

Prominent explanations of mutual exclusivity suppose that the child discriminates between objects on the basis of whether or not they can name an object. However, in the standard test of mutual exclusivity, the name-unknown objects are almost always more *novel* than the name-known objects. Hence, there is a fundamental confound between lexical knowledge and object familiarity. Either of these attributes could guide the child toward mapping a novel label with a novel, name-unknown object. There are several reasons to think that object novelty could play a role. First, outside of the laboratory, it might be easier for the child to detect new objects than to systematically retrieve names for the many objects in their surroundings. Second, it is well-established that even very young infants will habituate and preferentially attend to novel stimuli (e.g., Slater et al., 1982, 1983). Finally, there is a small, but growing body of evidence suggesting that object novelty does influence the mutual exclusivity response. Originally, studies by Merriman and colleagues (e.g., Merriman and Bowman, 1989; Merriman and Schuster, 1991), showed that 2-year-olds prefer to select the most novel object in response to a novel label. Children will make this response even when the more novel object is in fact an unfamiliar exemplar of a name-known object category (Merriman and Bowman, 1989). More recently, Horst et al. (2011) and Mather and Plunkett (2012) further demonstrate that toddlers favor the most novel object as a referent, even though other name-unknown objects may be present.

A problem which has beset traditional “constraints and biases” explanations is that they often do not account for the cognitive processes implementing a word-learning strategy. However, a more mechanistic account of mutual exclusivity is possible if we draw upon what we already understand about learning, memory, and attention. Novelty plays a fundamental role in all of these cognitive processes. Thus, clarifying the role of novelty in mutual exclusivity is an important first step toward understanding the underlying cognitive mechanism. However, given the surprisingly small amount of research into the effects of novelty on word learning, some important questions remain unanswered. Here I briefly discuss some of these issues in turn.

#### Is there a role for object names?

While there is a growing body of evidence for the effects of novelty on word learning, it is not yet clear that object novelty *alone* can account for the mutual exclusivity response. In Mather and Plunkett (2012), infants' speed of word mapping varied across experiments, according to whether or not a name-known object was present. Infants' mapping of a novel label to a novel object was more rapid when a name-known object was present. One possibility is that the infants' search for a novel referent was prompted more rapidly by the presence of a name-known object. Similarly, other patterns of response suggest that knowledge of object names might impact on the mutual exclusivity response. Halberda (2006) provides evidence that preschoolers and adults engage in a “double-check” of a familiar, name-known object when they hear a new word, even if they are already attending to a novel object. Findings from Halberda (2003) suggest that a similar process might operate for younger word-learners, except that younger infants might struggle with the process of excluding the familiar object as a referent. Thus, while science favors the most parsimonious explanation, it is yet possible that both novelty and object names impact on the mutual exclusivity response.

#### How does novelty impact on word learning?

Word-learning is a multi-stage process. Attention to the correct pairing of label and object does not mean an association has been learned (see McMurray et al., 2012). Thus, beyond the initial disambiguation, a word mapping has to be successfully encoded and retained over time. What role does novelty play at each of these stages? The evidence suggests that novelty directs the child's attention to the correct pairing of label and referent. Yet, while novelty may support disambiguation, it also increases processing load (e.g., Mather et al., 2011). How might this impact on encoding? If both label and object are novel, then the child has to simultaneously learn about the label, the object, and the association between the two. One issue concerns how processing resources are allocated across modalities. Research into the “auditory dominance” effect suggests that infants prioritize auditory processing at the expense of visual processing (e.g., Robinson and Sloutsky, 2004). Hence, novelty might selectively disrupt specific aspects of the encoding process. Encoding could also impact on subsequent retention. Few studies have tested for the retention of word mappings following a mutual exclusivity task. Bion et al. (2013) tested 18–30-month-olds for retention immediately following training, and found retention performance was related to attention during training to the novel referent (cf. Mather and Plunkett, 2011) However, Horst and Samuelson (2008) tested 2-year-olds for retention after a five-minute interval, and found that retention was poor. More research is necessary to understand how novelty impacts on encoding and the retention process. A final point is that new words are also consolidated and integrated with existing lexical knowledge, at least in older children and adults (e.g., Henderson et al., 2012). While this process is not understood in younger word learners, the novelty (or familiarity) of words and referents could plausibly influence this process.

#### The lure of the familiar?

Does the child always attend to novelty? A fascinating, yet poorly understood, characteristic of infant attention is that attention to novelty is sometimes preceded by attention to familiarity. When an infant has a choice between exploring a familiar and novel stimulus, a familiarity preference is thought to occur when the infant has not yet processed the familiar stimulus in detail (Hunter and Ames, 1988). Thus, attention to novelty may require sufficient processing of a familiar stimulus. In some studies of mutual exclusivity, toddlers have displayed preferential attention to the *familiar* object prior to the presentation of a novel label (Halberda, 2003; White and Morgan, 2008). To understand the role of novelty, we need to understand when and how *familiarity* could bias attention during word learning. A successful mutual exclusivity response may depend on the child's ability to disengage and shift their attention from familiarity to novelty upon hearing a novel label (see Axelsson et al., 2012, for a related argument). A detailed consideration of variables which could influence these preferences (e.g., trial duration, stimulus repetition, etc.) might also resolve discrepancies between different published findings (cf. Bion et al., 2013).

### How does this mechanism develop?

If the mechanism underlying the mutual exclusivity response is not clear, then the developmental origin of mutual exclusivity is even more uncertain. Some researchers have offered tentative explanations, but these accounts are far from complete. The N3C principle is one of six word learning principles within the “Developmental Lexical Principles Framework” of Golinkoff et al. (1994). Within this framework, there are two tiers: the first tier consists of basic principles about the referential nature of words and the extendibility of labels to similar objects. The second tier contains more sophisticated constraints allowing the rapid acquisition of new words, such as the N3C principle. Notably, the second tier of principles is thought to develop by building on the first tier. Mervis and Bertrand (1994) provide evidence that use of the N3C principle is linked to the onset of exhaustive categorical sorting and the “vocabulary spurt” (cf. Markman et al., 2003). However, the exact developmental mechanisms by which the child acquires the N3C principle are not specified.

A more recent examination of the development of mutual exclusivity is provided by Halberda (2003). In this study, Halberda demonstrates changes in the response to a mutual exclusivity task between the ages of 14–17 months. Specifically, while 17-month-olds would increase attention to a novel object upon hearing a novel label, younger infants would either respond inconsistently as a group, or even increase attention to the name-known object. Halberda has argued that the infants are “ruling out” the name-known object before mapping the label to the novel object. However, younger infants get stuck on this first step of excluding the familiar object. While this account illuminates the behavior of infants on the threshold of using mutual exclusivity, it does not reach back far enough to explain how infants learn to exclude the name-known object in the first place.

If novelty underlies the mutual exclusivity response, it might also influence the acquisition of mutual exclusivity. Indeed, infants as young as 10 months are sensitive to the novelty of objects and labels, even though they are far from being proficient word learners (Mather and Plunkett, 2010). Infants of this age may be displaying a “precursor” to mutual exclusivity, which develops into the full word-learning strategy at a later age. A viable possibility is that mutual exclusivity may emerge from attention to the novelty of objects and labels. However, we must also consider the effects of the learning environment. For example, bilingual infants do not display mutual exclusivity at the age at which it appears in monolingual infants (Houston-Price et al., 2010). Thus, attention to novelty, in interaction with the structure of the (monolingual) linguistic environment, may lead to the emergence of mutual exclusivity.

## Conclusions

Explicit teaching may not always be required to map words onto the world. Mutual exclusivity is a form of bootstrapping where the child uses existing knowledge to create new word meanings. However, precisely what information is exploited by the young word learner? Is it lexical knowledge or mere novelty? The evidence suggests that novelty matters—so we need to explore further how novelty influences word learning. Part of the solution will be to better understand the processing of novelty itself. When and why does the child shift attention from familiarity to novelty? How does novelty influence cross-modal processing? How does novelty impact on longer-term memory? Furthermore, these dimensions of novelty processing are likely to change with age, and may interact with the linguistic environment and the child's vocabulary development. A truly developmental approach may prove critical to understanding the mechanism(s) underlying mutual exclusivity.

## References

[B1] Axelsson E. L. Churchley K. Horst J. S. (2012). The right thing at the right time: why ostensive naming facilitates word learning. Front. Psychology 3:88 10.3389/fpsyg.2012.00088 22470363 PMC3314248

[B2] Bion R. A. H. Borovsky A. Fernald A. (2013). Fast mapping, slow learning: Disambiguation of novel word-object mappings in relation to vocabulary learning at 18, 24, and 30 months. Cognition 126, 39–53 10.1016/j.cognition.2012.08.008 23063233 PMC6590692

[B3] Bloom P. (2000). How Children Learn the Meanings of Words. Cambridge, MA: MIT Press

[B4] Clark E. (1987). The principle of contrast: a constraint on language acquisition, in Mechanisms of Language Acquisition, ed MacWhinney B. (Hillsdale, NJ: Lawrence Erlbaum Associates), 1–33

[B5] Golinkoff R. M. Mervis C. B. Hirsh-Pasek K. (1994). Early object labels– the case for a developmental lexical principles framework. J. Child Lang. 21, 125–155 8006089 10.1017/s0305000900008692

[B6] Halberda J. (2003). The development of a word-learning strategy. Cognition 87, B23–B34 10.1016/S0010-0277(02)00186-5 12499109

[B7] Halberda J. (2006). Is this a dax which I see before me? Use of the logical argument disjunctive syllogism supports word-learning in children and adults. Cogn. Psychol. 53, 310–344 10.1016/j.cogpsych.2006.04.003 16875685

[B8] Henderson L. M. Weighall A. R. Brown H. Gaskell M. G. (2012). Consolidation of vocabulary is associated with sleep in children. Dev. Sci. 15, 674–687 10.1111/j.1467-7687.2012.01172.x 22925515

[B9] Horst J. S. Samuelson L. K. (2008). Fast mapping but poor retention by 24-month-old infants. Infancy 13, 128–157 10.1080/15250000701795598 33412722

[B10] Horst J. S. Samuelson L. K. Kucker S. C. McMurray B. (2011). What's new? Children prefer novelty in referent selection. Cognition 118, 234–244 10.1016/j.cognition.2010.10.015 21092945 PMC3022084

[B11] Houston-Price C. Caloghiris Z. Raviglione E. (2010). Language experience shapes the development of the mutual exclusivity bias. Infancy 15, 125–150 10.1111/j.1532-7078.2009.00009.x 32693477

[B12] Hunter M. A. Ames E. W. (1988). A multifactor model of infant preferences for novel and familiar stimuli, in Advances in Infancy Research, Vol. 5, eds Rovee-Collier C. Lipsitt L. P. (Stamford, CT: Ablex), 69–95

[B13] Markman E. M. (1989). Categorization and Naming in Children: Problems of Induction. Cambridge, MA: MIT Press

[B14] Markman E. M. (1990). Constraints children place on word meanings. Cogn. Sci. 14, 57–77

[B15] Markman E. M. Wasow J. L. Hansen M. B. (2003). Use of the mutual exclusivity assumption by young word learners. Cogn. Psychol. 47, 241–275 10.1016/S0010-0285(03)00034-3 14559217

[B16] Mather E. Plunkett K. (2010). Novel labels support 10-month-olds' attention to novel objects. J. Exp. Child Psychol. 105, 232–242 10.1016/j.jecp.2009.11.004 20031152

[B17] Mather E. Plunkett K. (2011). Mutual exclusivity and phonological novelty constrain word learning at 16 months. J. Child Lang. 38, 933–950 10.1017/S0305000910000401 21092371

[B18] Mather E. Plunkett K. (2012). The role of novelty in early word learning. Cogn. Sci. 36, 1157–1177 10.1111/j.1551-6709.2012.01239.x 22436081

[B19] Mather E. Schafer G. Houston-Price C. (2011). The impact of novel labels on visual processing during infancy. Br. J. Dev. Psychol. 29, 783–805 10.1348/2044-835X.002008 21199503

[B19a] McMurray B. Horst J. S. Samuelson L. K. (2012). Word learning emerges from the interaction of online referent selection and slow associative learning. Psychol. Rev. 119, 831–877 10.1037/a0029872 23088341 PMC3632668

[B20] Merriman W. E. Bowman L. L. (1989). The mutual exclusivity bias in children's word learning. Monogr. Soc. Res. Child Dev. 54, 1–129 2608077

[B21] Merriman W. E. Schuster J. M. (1991). Young children's disambiguation of object name reference. Child Dev. 62, 1288–1301 1786716

[B22] Mervis C. B. Bertrand J. (1994). Acquisition of the novel name-nameless category (n3c) principle. Child Dev. 65, 1646–1662 7859547 10.1111/j.1467-8624.1994.tb00840.x

[B23] Quine W. V. O. (1960). Word and Object. Cambridge, MA: MIT Press

[B24] Robinson C. W. Sloutsky V. M. (2004). Auditory dominance and its change in the course of development. Child Dev. 75, 1387–1401 10.1111/j.1467-8624.2004.00747.x 15369521

[B25] Samuelson L. K. Smith L. B. (2000). Grounding development in cognitive processes. Child Dev. 71, 98–106 10.1111/1467-8624.00123 10836563

[B26] Slater A. Morison V. Rose D. (1982). Visual memory at birth. Br. J. Psychol. 73, 519–525 7171927 10.1111/j.2044-8295.1982.tb01834.x

[B27] Slater A. Morison V. Rose D. (1983). Locus of habituation in the human newborn. Perception 12, 593–598 6676710 10.1068/p120593

[B28] White K. S. Morgan J. L. (2008). Sub-segmental detail in early lexical representations. J. Mem. Lang. 59, 114–132

